# Feasibility of bronchial wall quantification in low‐ and ultralow‐dose third‐generation dual‐source CT: An ex vivo lung study

**DOI:** 10.1002/acm2.13032

**Published:** 2020-09-29

**Authors:** Lin Zhang, Gert Jan Pelgrim, Jing Yan, Hao Zhang, Rozemarijn Vliegenthart, Xueqian Xie

**Affiliations:** ^1^ Radiology Department Shanghai General Hospital Shanghai Jiao Tong University School of Medicine Shanghai China; ^2^ Radiology Department Shanghai General Hospital of Nanjing Medical University Shanghai China; ^3^ Department of Radiology University of Groningen University Medical Center Groningen Groningen The Netherlands; ^4^ Siemens Healthcare Ltd Shanghai China

**Keywords:** airway quantification, chronic obstructive pulmonary disease, radiologic phantom, radiation dosage, X‐ray computed tomography

## Abstract

**Purpose:**

To investigate image quality and bronchial wall quantification in low‐ and ultralow‐dose third‐generation dual‐source computed tomography (CT).

**Methods:**

A lung specimen from a formerly healthy male was scanned using third‐generation dual‐source CT at standard‐dose (51 mAs/120 kV, CTDI_vol_ 3.41 mGy), low‐dose (1/4th and 1/10th of standard dose), and ultralow‐dose setting (1/20th). Low kV (70, 80, 90, and Sn100 kV) scanning was applied in each low/ultralow‐dose setting, combined with adaptive mAs to keep a constant dose. Images were reconstructed at advanced modeled iterative reconstruction (ADMIRE) levels 1, 3, and 5 for each scan. Bronchial wall were semi‐automatically measured from the lobar level to subsegmental level. Spearman correlation analysis was performed between bronchial wall quantification (wall thickness and wall area percentage) and protocol settings (dose, kV, and ADMIRE). ANOVA with a post hoc pairwise test was used to compare signal‐to‐noise ratio (SNR), noise and bronchial wall quantification values among standard‐ and low/ultralow‐dose settings, and among ADMIRE levels.

**Results:**

Bronchial wall quantification had no correlation with dose level, kV, or ADMIRE level (|correlation coefficients| < 0.3). SNR and noise showed no statistically significant differences at different kV in the same ADMIRE level (1, 3, or 5) and in the same dose group (*P* > 0.05). Generally, there were no significant differences in bronchial wall quantification among the standard‐ and low/ultralow‐dose settings, and among different ADMIRE levels (*P* > 0.05).

**Conclusion:**

The combined use of low/ultralow‐dose scanning and ADMIRE does not influence bronchial wall quantification compared to standard‐dose CT. This specimen study suggests the potential that an ultralow‐dose scan can be used for bronchial wall quantification.

**Abbreviations**
CT: computed tomography
COPD: chronic obstructive pulmonary disease
ALARA: as‐low‐as‐reasonably achievable
kV: kilovolt
IR: iterative reconstruction
ADMIRE: advanced modeled iterative reconstruction
SNR: signal‐to‐noise ratio
CTDI_vol_: CT volume dose index
%WA: wall area percentage
WT: wall thickness
ROI: region of interest

## 1 INTRODUCTION

Airway quantification on computed tomography (CT) provides an estimate of bronchial remodeling and inflammation, which is associated with physiological parameters and symptoms in chronic obstructive pulmonary disease (COPD).^1^, ^2^, ^3^, ^4^, ^5^, ^6^ The increasing concern on radiation exposure in clinical practice stimulates the new techniques to decrease radiation dose,^7^ like peak kilovolt (kV) reduction and iterative reconstruction (IR).^8^ Iterative reconstruction has been widely used for reducing radiation dose while improving image quality.^9^, ^10^, ^11^, ^12^ Recently, due to the technical advances and natural tissue contrast between lung parenchyma and airway, the radiation dose of chest CT is possible to closer to that of chest x‐ray (approximately < 0.1 mSv).^13^, ^14^

Whether the lower radiation dose affects the evaluation of COPD compared with conventional‐dose CT has become an important issue in COPD studies. Some studies showed no significant side effect of dose reduction on emphysema evaluation.^15^, ^16^, ^17^ For example, ultralow‐dose CT at a radiation dose equivalent to 5% of the standard dose (2.33 ± 1.54 mSv), although increasing the percentage of low attenuation area, was strongly correlated with standard‐dose CT, thus could be used to evaluate lung volume and density.^15^ However, the reduction in radiation dose decreases the ability to display distal bronchi. Kirby et al. showed that the spatial resolution of 1‐2 mm in low‐dose CT (about 1.5 mSv) could identify the bronchi with an inner diameter greater than 2.5 mm.^18^ While CT examination at higher radiation dose (11.2 mSv) could show small airways with a diameter of 0.8 mm.^19^ In an ex vivo porcine lung specimen study when radiation dose decreased, the number of evaluable bronchial branches decreased, but measurement variability increased.^20^ To the best of our knowledge, there are few studies investigating bronchial wall quantification using low/ultralow‐dose CT in the human lung.

Low kV settings and advanced modeled iterative reconstruction (ADMIRE) have been recently introduced, available on third‐generation dual‐source CT systems.^21^ Therefore, this study aimed to comprehensively validate the correlation between bronchial wall quantification and low/ultralow‐dose CT techniques, using an ex vivo human lung specimen.

## 2 MATERIALS AND METHODS

### 2.A Specimen

An ex vivo lung from a formerly healthy nonsmoking male was used to evaluate the accuracy of bronchial measurements. The specimen size was about 19.5 cm long, 9.5 cm wide, and 7.5 mm high. The anatomy department of the medical school provided the specimen for research purposes.

### 2.B CT protocols

We used a third‐generation dual‐source CT (SOMATOM Force, Siemens Healthineers) for noncontrast single energy scanning. This study evaluated four different acquisition protocols with an estimated CT volume dose index (CTDI_vol_) ranging from 3.41 to 0.17 mGy. In the standard‐dose protocol, 120 kV and 110 mAs/51 mAs (target/effective mAs) were applied in accordance with standard clinical practice,^22^, ^23^, ^24^ resulting in CTDI_vol_ of 3.41 mGy. Three low/ultralow‐dose settings were used according to the pre‐defined CTDI_vol_, that is, 0.84 mGy (low‐dose: 1/4th of the standard dose), 0.33 mGy (low‐dose: 1/10th), and 0.17 mGy (ultralow‐dose: 1/20th). In each low‐ and ultralow‐dose setting, low kVs (70, 80, 90, and Sn100 kV) were applied. The Sn setting involves the prefiltration of the X‐ray beam by using a tin filter. This filter limits the range of the X‐ray energy spectrum reaching the scanned object.^25^ The tube current was adjusted to fit the predefined CTDI_vol_ for each tube voltage (Table 1). Other settings were kept constant across standard‐ and low/ultralow‐dose scans: matrix size 512 × 512, CARE Dose4D on, detector collimation 192 × 0.6 mm, rotation time 0.5 s (a slower gantry rotation speed for higher image quality),^26^ slice thickness 0.6 mm, and field of view 177 mm (cover all the specimen). All the images were reconstructed using Qr40 kernel (a kernel especially for quantitative analysis) with ADMIRE levels 1, 3, and 5, including the full‐dose scan. We repeated each scan five times and performed a small translocation and rotation of the specimen in between to simulate variability among repeated clinical scans. Due to certain differences between the specimen and human lung, a few of the scans could not be reconstructed by software, therefore, airway data of the corresponding repeated scan were excluded from the analysis, while SNR and noise data were retained.

**Table 1:** Predefined CTDI_vol_ and the corresponding multiple low‐kV and mAs settings.

Radiation dose	kV	mAs (target/effective)	CTDI_vol_ (mGy)
Standard‐dose setting	120	110/51	3.41
1/4th low‐dose setting	70	320/71	0.84
1/4th low‐dose setting	80	150/44	0.84
1/4th low‐dose setting	90	83/29	0.84
1/4th low‐dose setting	Sn100	550/248	0.84
1/10th low‐dose setting	70	135/28	0.33
1/10th low‐dose setting	80	62/17	0.33
1/10th low‐dose setting	90	35/11	0.33
1/10th low‐dose setting	Sn100	223/96	0.33
1/20th low‐dose setting	70	70/14	0.17
1/20th low‐dose setting	80	10/10 (minimum)	0.17
1/20th low‐dose setting	90	10/10 (minimum)	0.26 (excluded)^a^
1/20th low‐dose setting	Sn100	113/47	0.17

CTDI_vol_ denotes computed tomography volume dose index.

^a^ Because of the limitation of scanning parameter setting of Force CT, the minimum target mAs under 90 kV can only be set to 10 mAs, and the corresponding CTDI_vol_ was 0.26 mGy, which cannot reach 1/20th low‐dose setting, so we excluded the 90 kV at 1/20th low‐dose setting.

### 2.C Evaluation method

**Fig. 1:**
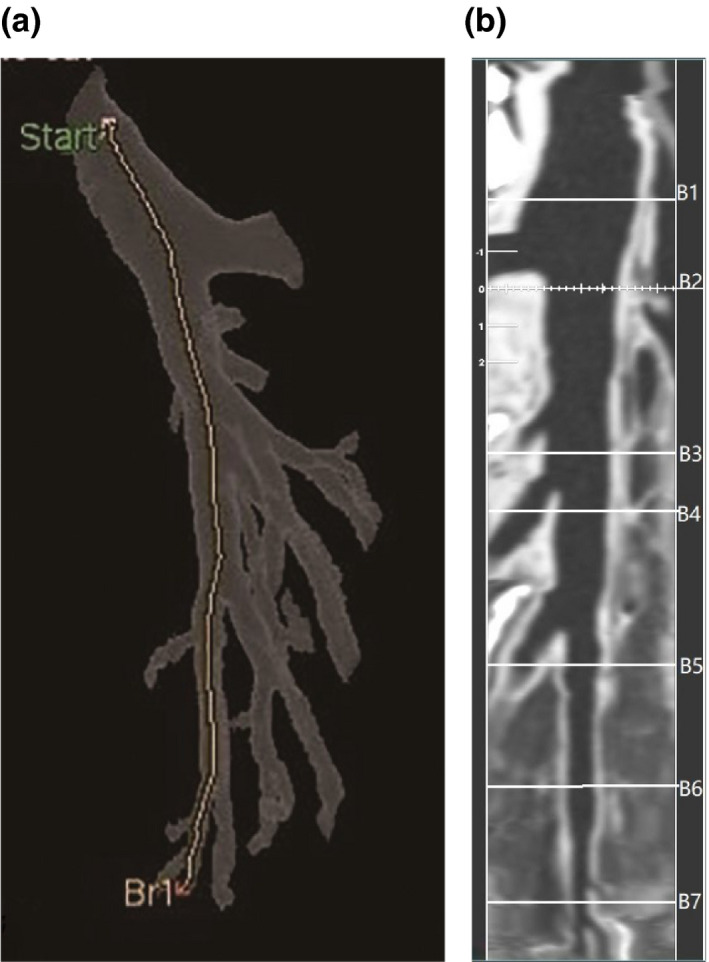
(a) Three‐dimensional reconstructed image illustrating the bronchial tree of the posterobasal segment lower lobe bronchus with the centerline. (b) Straightened rendering image along the centerline. B1 is the proximal section of the left lower lobe bronchus. B2–B7 are the proximal measurable sections of each successive segment downward bronchi of the posterobasal segment of the left lower lobe.

**Fig. 2:**
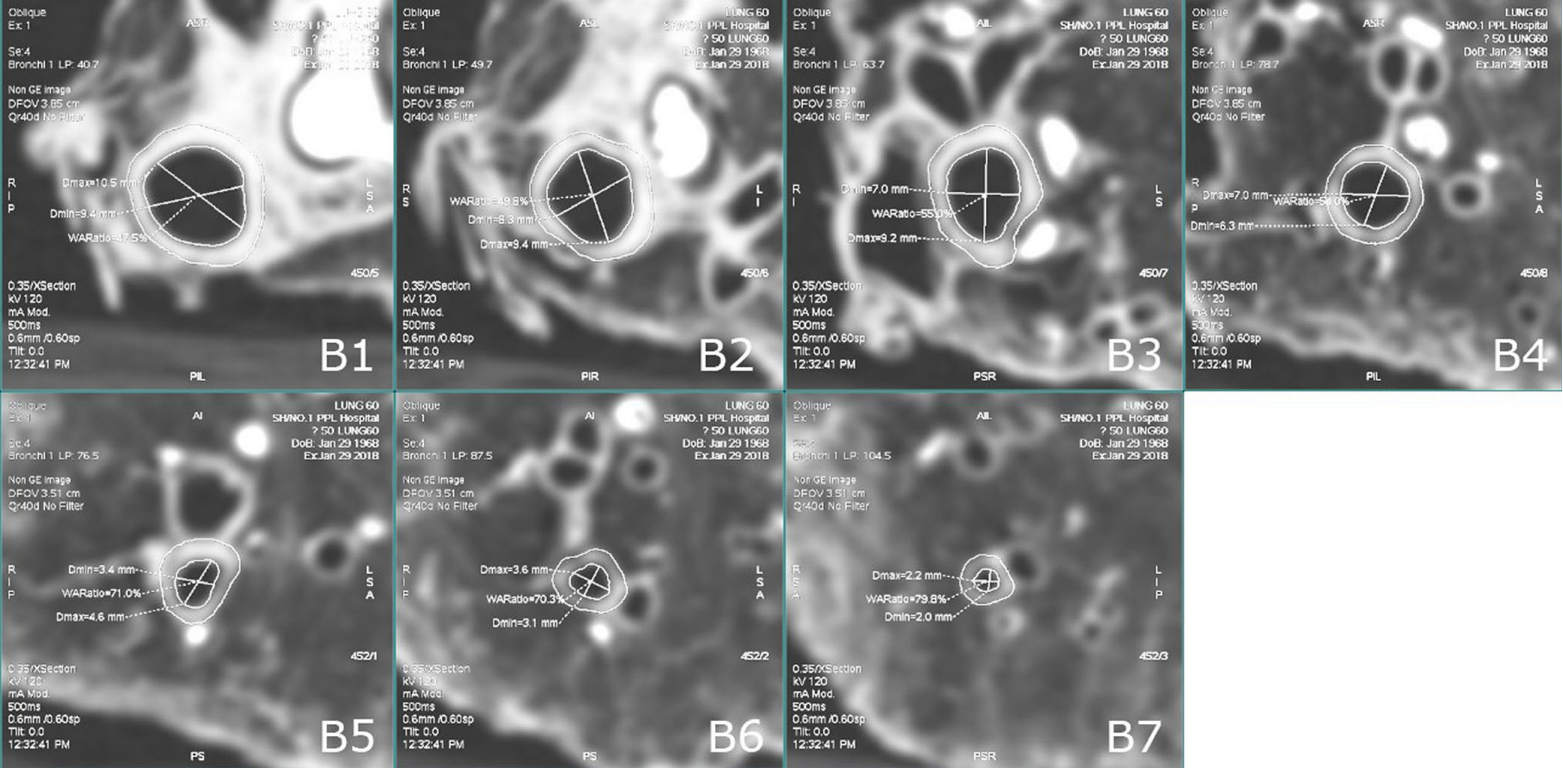
Measurement points of B1–B7 cross‐sectional images explained how the software segmented and measured wall thickness (WT) and wall area percentage (%WA).

A semi‐automated software (Thoracic VCAR, Advanced workstation 4.6, GE Healthcare) was used to perform bronchial wall measurement. A radiologist of 14 yr of experience who was unaware of the scan purpose and settings analyzed all images. The analyzing time interval between repeated scans was at least 15 days to avoid reader bias. We chose two points from the bronchial tree (the left main bronchus, and the distal of the posterobasal segmental lower lobe bronchus) to obtain a straightened image of the bronchus [Fig. 1(a)]. Then we selected seven points representing different diameters to outline the contour of the bronchus in the cross‐sectional images, and the cross‐section of the measured bronchus should be close to a round shape. B1 was in the left lower lobe bronchi, B2 in the next generation one third of the proximal bronchi, and so on. We marked the location of B1‐B7 in Fig. 1(b). The software automatically measured average wall thickness (WT) and wall area percentage (%WA). %WA was defined as (wall area)/(wall area + lumen area) × 100%. We showed multiple representative cross‐sectional images explaining how to segment and measure the bronchial wall in Fig. 2.

The measurement points across different scans were the same distance between the point and the left main bronchus bifurcation. Background signal‐to‐noise ratio (SNR) and noise were measured by placing a circular region of interest (ROI; area of 20 mm^2^) in the lumen of the left main bronchus.

### 2.D Statistical analysis

The relationship between measurement values (SNR, noise, WT, and %WA) and protocol settings was evaluated by Spearman correlation analysis. The protocol settings consisted of radiation dose levels (3.41, 0.86, 0.33, and 0.17 mGy), kV settings (70, 80, 90, and Sn100 kV), and ADMIRE levels (1, 3, and 5). Image SNR and noise among the standard‐ and three low/ultralow‐dose settings, among different kV settings, and among different ADMIRE levels were compared using multivariate ANOVA. The difference of bronchial measurements (WT and %WA) among dose settings, kV settings, and ADMIRE levels were pairwisely evaluated using the Student–Newman–Keuls post hoc test. Agreement between each of the low/ultralow‐dose settings and the standard‐dose setting was evaluated using Bland–Altman analysis, in which the value of the standard‐dose setting was subtracted from the low/ultralow‐dose setting and then divided by the mean. Two statistical analysis packages were used (MedCalc 15.8, MedCalc Software and SPSS 19.0, IBM). A value of *P* < 0.05 was considered statistically significant.

## 3 RESULTS

### 3.A Overview

**Fig. 3:**
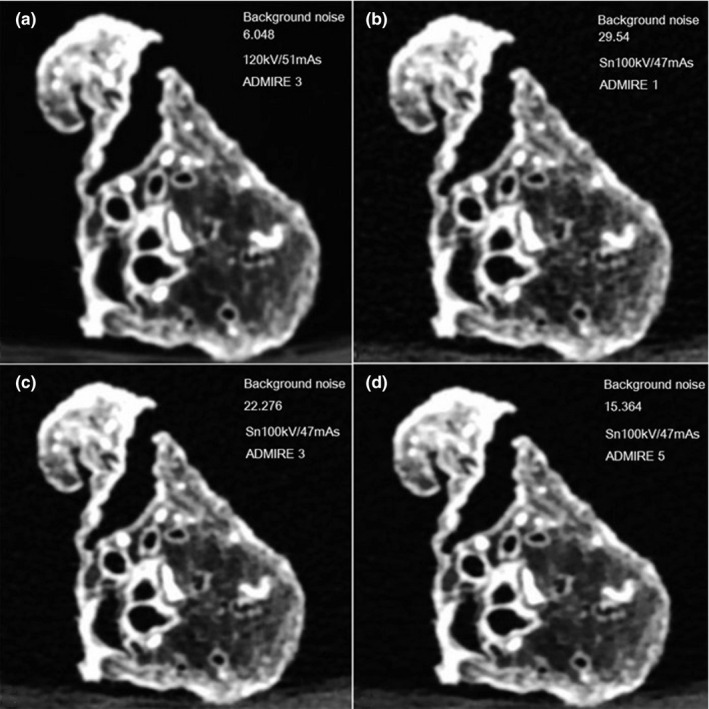
Axial images of the lung specimen. (a) Standard‐dose 120 kV/51 mAs, advanced modeled iterative reconstruction (ADMIRE) level 3. (b) 1/20 low‐dose Sn100 kV/47 mAs, ADMIRE 1. (c) 1/20 low‐dose Sn100 kV/47 mAs, ADMIRE 3. (d) 1/20 low‐dose Sn100 kV/47 mAs, ADMIRE 5. In these images, no blocky appearance in the high ADMIRE‐level image. The 1/20 low‐dose setting significantly increased the background noise compared to the standard dose. By using a higher ADMIRE level, the image noise was greatly compensated.

In total, 108 sets of image reconstruction were accomplished. All the images did not show blocky pixelated at any ADMIRE level (Fig. 3). The semi‐automated software accurately segmented the external and internal contours of the bronchial wall, without the need for manual editing. From the proximal to the distal airway, mean WT decreased from 2. 0 to 1.3 mm and mean %WA increased from 49.8% to 81.3%.

### 3.B Influential factors on SNR, image noise, and bronchial wall quantification

SNR was significantly correlated with dose level, and ADMIRE level (correlation coefficient 0.716 and 0.546, respectively, *P* < 0.001), but not with kV (0.219, *P* = 0.003). Image noise was significantly correlated with dose level, and ADMIRE level (−0.716 and −0.546, *P* < 0.001), but not with kV (−0.215, *P* = 0.004). WT and %WA had generally no correlation with dose level, kV, or ADMIRE level (the absolute values of correlation coefficients < 0.3), and the details are shown in Table S1.

### 3.C Pairwise comparisons of SNR and noise between dose settings, tube voltage settings, and ADMIRE levels

SNR and noise showed no statistically significant differences at different kV in the same ADMIRE level (1, 3, or 5) and in the same dose group (all *P* > 0.05). Details are shown in Tables S2 and S3.

Pairwise comparisons of SNR and noise of the four dose settings are shown in Table 2, where measures of kV settings in the same low/ultralow‐dose scans without significant difference in variance homogeneity were pooled. Compared with the standard‐dose, SNR reduction was significant in 1/4th, 1/10th, and 1/20th dose setting (all *P* < 0.001). However, the SNR difference between 1/10th and 1/20th dose group was not significant at ADMIRE 3 and 5 (*P* = 0.058 and *P* = 0.116, respectively). There was a significant noise increase with decreasing CT dose (*P* < 0.001), the only exception being between standard‐dose and 1/4th dose setting at ADMIRE level 5 (*P* = 0.062).

**Table 2:** Pairwise comparisons of signal‐noise‐ratio (SNR) and noise for low/ultralow‐dose settings versus standard‐dose setting

ADMIRE level	Mean ± SD				*P*‐value					
	Standard dose	1/4th dose	1/10th dose	1/20th dose	Standard vs 1/4th	Standard vs 1/10th	Standard vs 1/20th	1/4th vs 1/10th	1/4th vs 1/20th	1/10th vs 1/20th
SNR										
ADMIRE 1	121.13 ± 16.26	74.99 ± 13.13	45.56 ± 8.24	37.19 ± 7.08	<0.001	<0.001	<0.001	<0.001	<0.001	0.025
ADMIRE 3	167.57 ± 32.98	104.55 ± 19.93	62.08 ± 14.25	50.30 ± 12.31	<0.001	<0.001	<0.001	<0.001	<0.001	0.058
ADMIRE 5	242.02 ± 60.76	153.82 ± 34.03	91.21 ± 23.58	74.24 ± 22.19	<0.001	<0.001	<0.001	<0.001	<0.001	0.116
NOISE										
ADMIRE 1	8.24 ± 1.09	13.45 ± 2.23	22.10 ± 3.48	27.17 ± 4.54	0.003	<0.001	<0.001	<0.001	<0.001	<0.001
ADMIRE 3	6.05 ± 1.17	9.70 ± 1.79	16.43 ± 3.08	20.41 ± 4.10	0.016	<0.001	<0.001	<0.001	<0.001	<0.001
ADMIRE 5	4.28 ± 1.13	6.66 ± 1.42	11.32 ± 2.49	14.15 ± 3.66	0.062	<0.001	<0.001	<0.001	<0.001	0.002

Results are described as mean ± SD and *P*‐value.

SNR represents signal‐to‐noise ratio; ADMIRE represents advanced modeled iterative reconstruction; and SD represents standard deviation

**Fig. 4:**
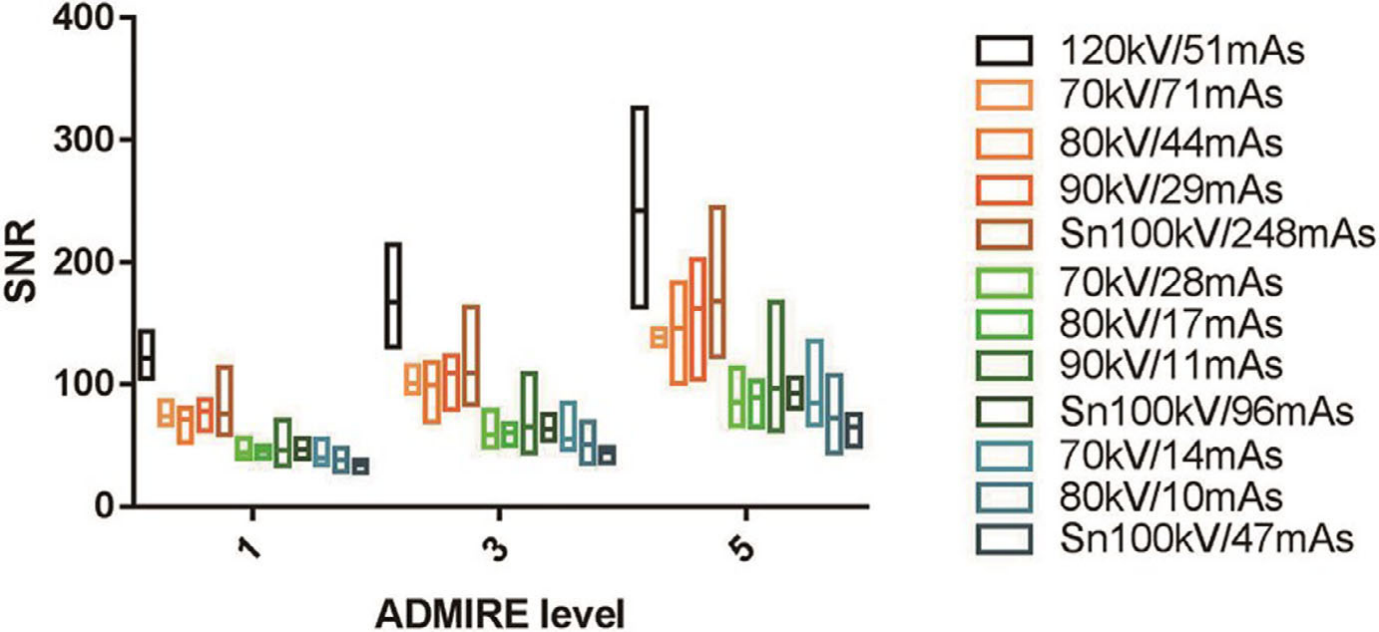
Signal‐to‐noise ratio (SNR) among different kV/mAs settings at advanced modeled iterative reconstruction (ADMIRE) levels 1, 3, and 5. SNR in ADMIRE 5 increased for all the kV/mAs settings. Using ADMIRE 5, Sn setting increased SNR in a low‐dose protocol (0.84 and 0.33 mGy), but reduced SNR in ultralow‐dose setting (0.17 mGy).

**Fig. 5:**
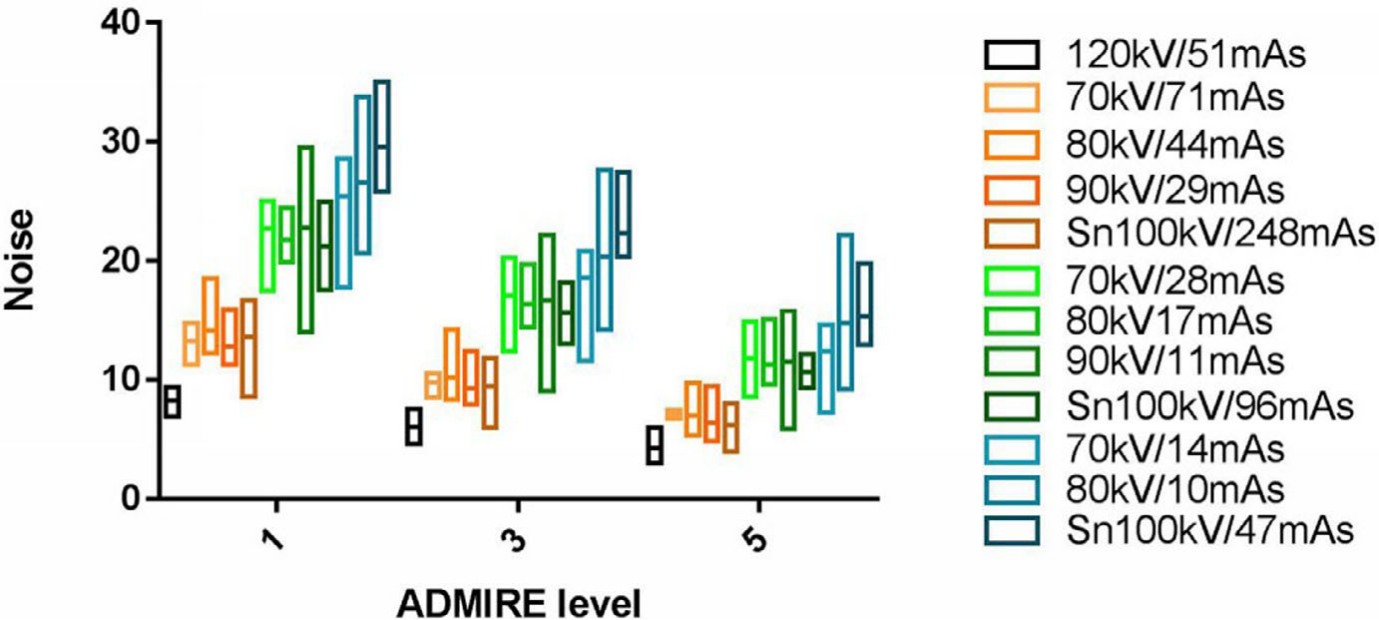
Image noise for different kV/mAs settings at advanced modeled iterative reconstruction (ADMIRE) levels 1, 3, and 5. Using ADMIRE 5, image noise was lower than that in ADMIRE 1 and 3. Using tin filtration increased image noise for 1/20th dose scans, while combined with higher mAs and higher ADMIRE level decreased in image noise.

SNR showed significant differences between ADMIRE 1 and 5 (*P* = 0.001), between ADMIRE 3 and 5 (*P* = 0.023), but not between ADMIRE 1 and 3 (*P* = 0.170; Fig. 4). Noise showed significant differences between ADMIRE 1 and 5 (*P* < 0.001), between ADMIRE 3 and 5 (*P* = 0.029), and between ADMIRE 1 and 3 (*P* = 0.014; Fig. 5). Details are shown in Appendix Table 4.

### 3.D Pairwise comparisons of bronchial wall quantification in different dose settings

There were no significant differences in WT and %WA between each of the low/ultralow‐dose settings and standard‐dose setting or in pairwise comparison for the different low/ultralow‐dose settings (all *P* > 0.05; Table 3).

**Table 3:** Pairwise comparisons of wall thickness (WT) and wall area percentage (%WA) for dose settings

kV settings	Mean ± SD				*P*‐value					
	Standard dose	1/4th dose	1/10th dose	1/20th dose	Standard vs 1/4th	Standard vs 1/10th	Standard vs 1/20th	1/4th vs 1/10th	1/4th vs 1/20th	1/10th vs 1/20th
WT (mm)										
70kV	1.71 ± 0.26	1.68 ± 0.25	1.70 ± 0.26	1.73 ± 0.29	0.463	0.815	0.664	0.617	0.243	0.505
80kV		1.71 ± 0.27	1.73 ± 0.26	1.71 ± 0.29	1.000	0.793	0.974	0.793	0.974	0.819
90kV		1.71 ± 0.25	1.73 ± 0.29	—	0.948	0.669	—	0.622	—	—
Sn100kV		1.72 ± 0.28	1.72 ± 0.27	1.74 ± 0.28	0.844	0.844	0.624	1.000	0.769	0.769
%WA										
70kV	62.37 ± 11.43	62.04 ± 11.23	62.45 ± 11.37	62.53 ± 11.25	0.869	0.969	0.939	0.839	0.809	0.970
80kV		62.53 ± 11.10	62.49 ± 11.34	62.65 ± 11.06	0.939	0.955	0.890	0.984	0.951	0.935
90kV		62.34 ± 11.43	62.68 ± 11.15	—	0.989	0.880	—	0.869	—	—
Sn100kV		62.40 ± 11.24	62.54 ± 11.07	62.79 ± 11.17	0.989	0.934	0.837	0.945	0.847	0.902

Results were described as mean ± SD and *P*‐value.

WT denotes wall thickness; %WA denotes wall area percentage; SD denotes standard deviation

### 3.E Agreement of bronchial wall quantifications between standard‐ and each of low/ultralow‐dose settings

Agreement of bronchial quantifications (WT and %WA) between standard‐dose and each low‐dose settings (1/4th, 1/10th, and 1/20th low‐dose) is shown in Table 4 by Bland–Altman analysis. For WT, the mean difference (95% CI) was −0.456% (−1.185%, 0.272%), 0.367% (−0.383%, 1.117%), 0.686% (−0.408%, 1.779%), respectively. For %WA, the mean difference was −0.026% (−0.365%, 0.313%), 0.316% (−0.045%, 0.677%), and 0.538% (−0.128%, 0.949%), respectively.

**Table 4:** Agreement of wall thickness (WT) and wall area percentage (%WA) between each of low/ultralow‐dose settings and standard‐dose setting evaluated by Bland–Altman analysis

	Plot differences as %			
	Arithmetic mean	1.96SD	95% CI	Plots outside the interval/total plots
%WA 1/4	−0.0263	−5.4, 5.3	−0.365, 0.313	13/252
%WA 1/10	0.316	−5.4, 6.0	−0.0446, 0.677	10/252
%WA 1/20	0.538	−5.1, 6.1	0.128, 0.949	13/189
WT 1/4	−0.457	−12.0, 11.1	−1.185, 0.272	9/252
WT 1/10	0.367	−11.5, 12.2	−0.383, 1.117	11/252
WT 1/20	0.686	−14.2, 15.6	−0.408, 1.779	7/189

WT represents wall thickness; %WA represents wall area percentage; SD represents standard deviation; and CI represents confidence interval.

### 3.F Pairwise comparisons of bronchial quantification in kV settings

There was no significant differences in WT and %WA in pairwise comparisons of the four kV settings, at the same low‐dose and same ADMIRE level (all *P* > 0.05). The detailed table is not shown because bronchial wall values did not correlate with kV settings.

### 3.G Pairwise comparisons of bronchial quantification at the three ADMIRE levels

In pairwise comparisons of WT, generally, no significant difference was observed, except for bronchus B2 between ADMIRE 1 and 5 (*P* = 0.019). In pairwise comparisons of %WA along B1 to B7 bronchi at ADMIRE levels 1, 3, and 5, no significant difference was observed, except for bronchus B2 and B6 between ADMIRE 1 and 5 (*P* < 0.05). There was no significant differences in WT and %WA between ADMIRE 1 and 3 or between ADMIRE 3 and 5 (all *P*> 0.05). Measures at different doses and kV settings were pooled.

## 4 DISCUSSION

Low‐dose chest CT has been clinically implemented for more than 20 yr.^27^ Recently introduced ultralow‐dose CT with a radiation dose similar to chest X‐ray (approximately 0.15 mGy or 0.06 mSv) has been applied to pulmonary diseases.^14^, ^17^, ^28^ Studies for low/ultralow‐dose CT mainly focused on validation of image quality,^13^, ^23^, ^24^, ^29^ disease detection, and dose reduction.^25^, ^30^ Its application in thoracic imaging mainly on diagnostic confidence and detectability of pulmonary nodules,^13^, ^14^, ^22^, ^28^, ^31^, ^32^ only a few on interstitial pulmonary disease,^24^ pulmonary inflammation,^14^ emphysema evaluation^17^, and airway assessment.^20^, ^33^ The growing morbidity and mortality of COPD has given rise to suggestions for screening using low/ultralow‐dose CT in the at‐risk population for preventive treatment,^34^ and to identify specific subgroups and exacerbation which may be amenable to therapy.^1^ Therefore, we studied the image quality using low/ultralow‐dose CT, low kV settings, and ADMIRE levels, and analyzed the impact on bronchial wall quantification. We found that SNR and noise were significantly influenced by dose levels and ADMIRE levels, but not significantly influenced by kV settings. Dose, kV settings, and ADMIRE levels showed no obvious influence on bronchial wall quantification.

Radiation dose, image quality, and diagnostic accuracy in low/ultralow‐dose CT need to be balanced. There is a disagreement on whether low/ultralow‐dose scanning affects CT measurements such as emphysema index,^16^, ^17^ related to reduced image quality. A recent study showed that advanced CT techniques like tin filtration and IR were able to generate CT images with acceptable noise for quantification at ultralow‐dose (CTDI_vol_ of 0.15 mGy) in COPD patients.^13^ For airway measurements, an ex vivo study showed that a low radiation dose (minimum of 0.25 mGy) did not influence measured airway parameters using IR.^20^ An in vivo large animal study showed that ultralow‐dose CT protocols had small measurement differences of %WA and WT in small airways.^33^ In our study, similar to previous results,^20^, ^33^, ^35^ %WA and WT were not significantly influenced by dose reduction, although the variability of measurements slightly increased. Due to acceptable image quality in ultralow‐dose setting, good contrast between the bronchial wall and adjacent lung tissue could help to obtain airway measurements close to the standard dose. Very few outliers may be explained by a lack of good tissue contrast affecting image segmentation in the proximal bronchus.

ADMIRE allows to decrease radiation exposure by retrospectively eliminating increased image noise, and therefore retaining image quality.^23^, ^30^ The ADMIRE level mainly controls the strength of noise reduction. Therefore, an increasing ADMIRE level should allow higher radiation dose reduction.^9^ In our study, ADMIRE 5 resulted in improved SNR and lower noise in comparison to ADMIRE 1 and 3 with the same radiation dose, which confirms results from previous studies.^13^, ^36^ Although the ADMIRE strength of 5 possibly had a higher amount of noise reduction, blocky appearance (losing detail in the image) of higher strength IR^30^, ^37^ was not observed in our study, which may due to ADMIRE’s excellent noise reduction potential, the technology adopts a "statistical model" and iterative decoding chip to integrate the statistical data of virtual data domain, image domain, and model domain efficiently.^38^ Through multiple iterations, the artifact was removed and the noise was reduced to achieve real‐time high‐definition iterative imaging.^38^ Similar results could be found in the latest IR reconstruction studies of other venders.^39^, ^40^ According to our results, interestingly, the higher ADMIRE level was associated with higher image SNR and lower image background noise. For the measurement of the airway, we found that the ADMIRE level had no significant influence on the measured values, which were in accordance with Leutz‐Schmidt’s study.^20^ Furthermore, the measurement variability of %WA and WT was acceptable in the three low/ultralow‐dose settings.

Low kV and spectral filtration allow dose reduction, but their effect on image quality and bronchial wall measurements need to be studied. Different kVp selection in low/ultralow‐dose chest CT can be used, namely 70 kV,^23^, ^41^ 80 kV,^31^, ^42^ 90 kV,^43^ Sn100 kV,^28^, ^29^ and Sn150 kV.^28^ A previous study showed that an Sn100kV setting yielded better image quality in comparison with 70 to 90 kV protocols at similar dose levels in the parasinal region.^25^ In another anthropomorphic chest phantom study, Sn100 kV was found to be better than 70 kV for nodule detection and noise reduction in low/ultralow‐dose CT using ADMIRE 3 and 5.^28^ A study of 1/10 dose (0.32 mSv) using Sn100 kV showed that subjective image quality was not statistically significantly different from the standard 3 mSv dose group.^29^ In our study, image SNR and noise values and airway quantification values showed no obvious changes among kV settings at the same dose and the same ADMIRE level, although Fig. 4 showed a slight noise reduction in the 1/20 ultralow‐dose using Sn100kV and ADMIRE 5. In terms of diagnosis, low kV showed no significant difference from the standard dose in the bronchial wall measurement. We speculated that the result may be related to the scanned object.

This study has limitations. First, although the ex vivo lung lacks radiation absorption by the thoracic cage, similar human lung specimens for structural evaluation have proved its usability for research.^34^ We used CARE Dose4D to adjust tube current, and thus, exposure dose. Second, the measurements using standard dose were considered as the reference standard because it was impossible to dissect the lung specimen to obtain true values of the bronchial. Third, CTDI_vol_ is an indirect measure but a surrogate indicating the radiation output of the CT system.^21^ Fourth, our results need to be confirmed in clinical patient studies with regard to the impact on quantitative emphysema assessment before implementation in COPD patients.

## 5 CONCLUSION

Ultralow‐dose settings increased image noise and slightly increased measurement variability, combined higher ADMIRE compensated for the increased noise caused by low‐dose while did not significantly influence the bronchial measurements. This specimen study suggests that an ultralow‐dose scan as low as 0.17 mGy is useful for bronchial wall quantification. If a patient study confirms these findings, ultralow‐dose settings could be used in COPD patients.

## AUTHOR CONTRIBUTION STATEMENT

Lin Zhang MD PhD made substantial contributions to the conception of the work, acquisition, analysis, interpretation of data, and drafting the work. Gert Jan Pelgrim MD PhD revised it critically for important intellectual content. Jing Yan PhD offered important technical support on the scanning. Hao Zhang MD PhD gave important suggestion of the manuscript. Rozemarijn Vliegenthart MD PhD gave important suggestion on statistics, and revised it critically for important intellectual content. Xueqian Xie MD PhD made final approval of the version to be published, and agreed to be accountable for all aspects of the work in ensuring that questions related to the accuracy or integrity of any part of the work are appropriately investigated and resolved.

## CONFLICT OF INTEREST

The authors of this manuscript declare that they have no conflict of interest related to this study.

## Informed Consent and Ethical Approval

Written informed consent was waived by the Institutional Review Board. Institutional Review Board approval was obtained.

## Supporting information


**Data S1**. Online supplementary.

## References

[1] Lynch DA , Al‐Qaisi MA . Quantitative computed tomography in chronic obstructive pulmonary disease. J Thorac Imaging. 2013;28:284–290. 23748651 10.1097/RTI.0b013e318298733c PMC4161463

[2] Newell JD Jr , Sieren J , Hoffman EA . Development of quantitative computed tomography lung protocols. J Thorac Imaging. 2013;28:266–271. 23934142 10.1097/RTI.0b013e31829f6796 PMC3876949

[3] Dijkstra AE , Postma DS , ten Hacken N , et al. Low‐dose CT measurements of airway dimensions and emphysema associated with airflow limitation in heavy smokers: a cross sectional study. Respir Res. 2013;14:11. 23356533 10.1186/1465-9921-14-11 PMC3570364

[4] Xie X , de Jong PA , Oudkerk M , et al. Morphological measurements in computed tomography correlate with airflow obstruction in chronic obstructive pulmonary disease: systematic review and meta‐analysis. Eur Radiol. 2012;22:2085–2093. 22699870 10.1007/s00330-012-2480-8 PMC3431473

[5] Nambu A , Zach J , Schroeder J , et al. Quantitative computed tomography measurements to evaluate airway disease in chronic obstructive pulmonary disease: relationship to physiological measurements, clinical index and visual assessment of airway disease. Eur J Radiol. 2016;85:2144–2151. 27776670 10.1016/j.ejrad.2016.09.010 PMC5310933

[6] Sasaki T , Takahashi K , Takada N , Ohsaki Y . Ratios of peripheral‐to‐central airway lumen area and percentage wall area as predictors of severity of chronic obstructive pulmonary disease. AJR Am J Roentgenol. 2014;203:78–84. 24951198 10.2214/AJR.13.11748

[7] Brenner DJ , Hall EJ . Computed tomography–an increasing source of radiation exposure. N Engl J Med. 2007;357:2277–2284. 18046031 10.1056/NEJMra072149

[8] Padole A , Ali Khawaja RD , Kalra MK , Singh S . CT radiation dose and iterative reconstruction techniques. AJR Am J Roentgenol. 2015;204:W384–W392. 25794087 10.2214/AJR.14.13241

[9] Ellmann S , Kammerer F , Allmendinger T , et al. Advanced modeled iterative reconstruction (ADMIRE) facilitates radiation dose reduction in abdominal CT. Acad Radiol. 2018;25:1277–1284. 29500115 10.1016/j.acra.2018.01.014

[10] Gatti M , Marchisio F , Fronda M , et al. Adaptive statistical iterative reconstruction‐V versus adaptive statistical iterative reconstruction: impact on dose reduction and image quality in body computed tomography. J Comput Assist Tomogr. 2018;42:191–196. 28937493 10.1097/RCT.0000000000000677

[11] Choo JY , Goo JM , Lee CH , Park CM , Park SJ , Shim MS . Quantitative analysis of emphysema and airway measurements according to iterative reconstruction algorithms: comparison of filtered back projection, adaptive statistical iterative reconstruction and model‐based iterative reconstruction. Eur Radiol. 2014;24:799–806. 24275806 10.1007/s00330-013-3078-5

[12] Singh S , Kalra MK , Do S , et al. Comparison of hybrid and pure iterative reconstruction techniques with conventional filtered back projection: dose reduction potential in the abdomen. J Comput Assist Tomogr. 2012;36:347–353. 22592622 10.1097/RCT.0b013e31824e639e

[13] Newell JD Jr , Fuld MK , Allmendinger T , et al. Very low‐dose (0.15 mGy) chest CT protocols using the COPDGene 2 test object and a third‐generation dual‐source CT scanner with corresponding third‐generation iterative reconstruction software. Invest Radiol. 2015;50:40–45. 25198834 10.1097/RLI.0000000000000093 PMC4294320

[14] Martini K , Barth BK , Nguyen‐Kim TD , Baumueller S , Alkadhi H , Frauenfelder T . Evaluation of pulmonary nodules and infection on chest CT with radiation dose equivalent to chest radiography: Prospective intra‐individual comparison study to standard dose CT. Eur J Radiol. 2016;85:360–365. 26781141 10.1016/j.ejrad.2015.11.036

[15] O'Brien C , Kok HK , Kelly B , et al. To investigate dose reduction and comparability of standard dose CT vs Ultra low dose CT in evaluating pulmonary emphysema. Clin Imaging. 2019;53:115–119. 30340073 10.1016/j.clinimag.2018.10.012

[16] Yuan R , Mayo JR , Hogg JC , et al. The effects of radiation dose and CT manufacturer on measurements of lung densitometry. Chest. 2007;132:617–623. 17573501 10.1378/chest.06-2325

[17] Messerli M , Ottilinger T , Warschkow R , et al. Emphysema quantification and lung volumetry in chest X‐ray equivalent ultralow dose CT ‐ intra‐individual comparison with standard dose CT. Eur J Radiol. 2017;91:1–9. 28629554 10.1016/j.ejrad.2017.03.003

[18] Kirby M , Tanabe N , Tan WC , et al. Total airway count on computed tomography and the risk of chronic obstructive pulmonary disease progression. Findings from a population‐based study. Am J Respir Crit Care Med. 2018;197:56–65. 28886252 10.1164/rccm.201704-0692OC

[19] Kurashima K , Hoshi T , Takaku Y , et al. Changes in the airway lumen and surrounding parenchyma in chronic obstructive pulmonary disease. Int J Chron Obstruct Pulmon Dis. 2013;8:523–532. 24204134 10.2147/COPD.S52637 PMC3817018

[20] Leutz‐Schmidt P , Weinheimer O , Jobst BJ , et al. Influence of exposure parameters and iterative reconstruction on automatic airway segmentation and analysis on MDCT‐an ex vivo phantom study. PLoS One. 2017;12:e0182268. 28767732 10.1371/journal.pone.0182268 PMC5540604

[21] Solomon J , Mileto A , Ramirez‐Giraldo JC , Samei E . Diagnostic performance of an advanced modeled iterative reconstruction algorithm for low‐contrast detectability with a third‐generation dual‐source multidetector CT scanner: potential for radiation dose reduction in a multireader study. Radiology. 2015;275:735–745. 25751228 10.1148/radiol.15142005

[22] Neroladaki A , Botsikas D , Boudabbous S , Becker CD , Montet X . Computed tomography of the chest with model‐based iterative reconstruction using a radiation exposure similar to chest X‐ray examination: preliminary observations. Eur Radiol. 2013;23:360–366. 22892722 10.1007/s00330-012-2627-7

[23] Burgard CA , Gaass T , Bonert M , et al. Detection of artificial pulmonary lung nodules in ultralow‐dose CT using an ex vivo lung phantom. PLoS One. 2018;13:e0190501. 29298331 10.1371/journal.pone.0190501 PMC5752031

[24] Braun FM , Johnson TR , Sommer WH , Thierfelder KM , Meinel FG . Chest CT using spectral filtration: radiation dose, image quality, and spectrum of clinical utility. Eur Radiol. 2015;25:1598–1606. 25515204 10.1007/s00330-014-3559-1

[25] Lell MM , May MS , Brand M , et al. Imaging the parasinus region with a third‐generation dual‐source CT and the effect of tin filtration on image quality and radiation dose. AJNR Am J Neuroradiol. 2015;36:1225–1230. 25814658 10.3174/ajnr.A4270 PMC7965271

[26] Beeres M , Wichmann JL , Paul J , et al. CT chest and gantry rotation time: does the rotation time influence image quality? Acta Radiol. 2015;56:950–954. 25140057 10.1177/0284185114544242

[27] Zwirewich CV , Mayo JR , Muller NL . Low‐dose high‐resolution CT of lung parenchyma. Radiology. 1991;180:413–417. 2068303 10.1148/radiology.180.2.2068303

[28] Gordic S , Morsbach F , Schmidt B , et al. Ultralow‐dose chest computed tomography for pulmonary nodule detection: first performance evaluation of single energy scanning with spectral shaping. Invest Radiol. 2014;49:465–473. 24598443 10.1097/RLI.0000000000000037

[29] Haubenreisser H , Meyer M , Sudarski S , Allmendinger T , Schoenberg SO , Henzler T . Unenhanced third‐generation dual‐source chest CT using a tin filter for spectral shaping at 100kVp. Eur J Radiol. 2015;84:1608–1613. 26001437 10.1016/j.ejrad.2015.04.018

[30] Schaller F , Sedlmair M , Raupach R , Uder M , Lell M . Noise reduction in abdominal computed tomography applying iterative reconstruction (ADMIRE). Acad Radiol. 2016;23:1230–1238. 27318787 10.1016/j.acra.2016.05.016

[31] Huber A , Landau J , Ebner L , et al. Performance of ultralow‐dose CT with iterative reconstruction in lung cancer screening: limiting radiation exposure to the equivalent of conventional chest X‐ray imaging. Eur Radiol. 2016;26:3643–3652. 26813670 10.1007/s00330-015-4192-3

[32] Vardhanabhuti V , Pang CL , Tenant S , Taylor J , Hyde C , Roobottom C . Prospective intra‐individual comparison of standard dose versus reduced‐dose thoracic CT using hybrid and pure iterative reconstruction in a follow‐up cohort of pulmonary nodules‐Effect of detectability of pulmonary nodules with lowering dose based on nodule size, type and body mass index. Eur J Radiol. 2017;91:130–141. 28629559 10.1016/j.ejrad.2017.04.006

[33] Hammond E , Sloan C , Newell JD Jr. , et al. Comparison of low‐ and ultralow‐dose computed tomography protocols for quantitative lung and airway assessment. Med Phys. 2017;44:4747–4757. 28657201 10.1002/mp.12436 PMC5603212

[34] Yanagawa M , Hata A , Honda O , et al. Subjective and objective comparisons of image quality between ultra‐high‐resolution CT and conventional area detector CT in phantoms and cadaveric human lungs. Eur Radiol. 2018;28:5060–5068. 29845337 10.1007/s00330-018-5491-2 PMC6223853

[35] Rodriguez A , Ranallo FN , Judy PF , Gierada DS , Fain SB . CT reconstruction techniques for improved accuracy of lung CT airway measurement. Med Phys. 2014;41:111911. 25370644 10.1118/1.4898098 PMC4241826

[36] Gordic S , Desbiolles L , Stolzmann P , et al. Advanced modelled iterative reconstruction for abdominal CT: qualitative and quantitative evaluation. Clin Radiol. 2014;69:e497–e504. 25239788 10.1016/j.crad.2014.08.012

[37] Barca P , Giannelli M , Fantacci ME , Caramella D . Computed tomography imaging with the adaptive statistical iterative reconstruction (ASIR) algorithm: dependence of image quality on the blending level of reconstruction. Australas Phys Eng Sci Med. 2018;41:463–473. 29737491 10.1007/s13246-018-0645-8

[38] Rompel OGM , Janka R , Dittrich S , et al. Third‐generation dual‐source 70‐kVp chest CT angiography with advanced iterative reconstruction in young children: image quality and radiation dose reduction. Pediatr Radiol. 2016;46:462–472. 26739141 10.1007/s00247-015-3510-x

[39] Zhang L , Li Z , Meng J , Xie X , Zhang H . Airway quantification using adaptive statistical iterative reconstruction‐V on wide‐detector low‐dose CT: a validation study on lung specimen. Jpn J Radiol. 2019;37:390–398. 30820822 10.1007/s11604-019-00818-2

[40] Benz DC , Grani C , Mikulicic F , et al. Adaptive statistical iterative reconstruction‐V: impact on image quality in ultralow‐dose coronary computed tomography angiography. J Comput Assist Tomogr. 2016;40:958–963. 27560012 10.1097/RCT.0000000000000460

[41] Weis M , Henzler T , Nance JW Jr. , et al. Radiation dose comparison between 70 kVp and 100 kVp with spectral beam shaping for non‐contrast‐enhanced pediatric chest computed tomography: a prospective randomized controlled study. Invest Radiol. 2017;52:155–162. 27662576 10.1097/RLI.0000000000000325

[42] Sui X , Meinel FG , Song W , et al. Detection and size measurements of pulmonary nodules in ultra‐low‐dose CT with iterative reconstruction compared to low dose CT. Eur J Radiol. 2016;85:564–570. 26860668 10.1016/j.ejrad.2015.12.013

[43] Leithner D , Gruber‐Rouh T , Beeres M , et al. 90‐kVp low‐tube‐voltage CT pulmonary angiography in combination with advanced modelled iterative reconstruction algorithm: effects on radiation dose, image quality, and diagnostic accuracy for the detection of pulmonary embolism. Br J Radiol. 2018;91:20180269. 29792729 10.1259/bjr.20180269 PMC6209482

