# Supplementary material for: Feasibility of bronchial wall quantification in low‐ and ultralow‐dose third‐generation dual‐source CT: An ex vivo lung study
Source: J Appl Clin Med Phys. 2020 Sep 29;21(10):218–26. doi: 10.1002/acm2.13032 (PMC7592972; doi:10.1002/acm2.13032)
Supplement: Supplementary file 1 — Data S1. Online supplementary. [file ACM2-21-218-s001.docx]

**Online supplementary**

**Appendix Table 1.** The correlation coefficients between wall thickness (WT) and wall area percentage (%WA) with dose level, kV or advanced modeled iterative reconstruction (ADMIRE) levels.

|  | **Correlation coefficients** | | | | | | |
| --- | --- | --- | --- | --- | --- | --- | --- |
|  | **%WA B1** | **%WA B2** | **%WA B3** | **%WA B4** | **%WA B5** | **%WA B6** | **%WA B7** |
| **Dose**  **kV**  **ADMIRE** | **-0.259*** | **-0.074** | **-0.135** | **-0.056** | **-0.042** | **-0.134** | **0.062** |
|  | **-0.103** | **0.248*** | **0.044** | **0.103** | **0.031** | **-0.011** | **0.199*** |
|  | **0.177** | **0.201*** | **-0.142** | **0.082** | **-0.050** | **-0.175** | **-0.066** |
|  | **WT B1** | **WT B2** | **WT B3** | **WT B4** | **WT B5** | **WT B6** | **WT B7** |
| **Dose**  **kV**  **ADMIRE** | **-0.223*** | **-0.128** | **-0.147** | **-0.023** | **-0.067** | **-0.185** | **0.071** |
|  | **0.070** | **0.150** | **0.059** | **0.371*** | **0.050** | **0.066** | **0.073** |
|  | **0.037** | **0.233*** | **-0.140** | **-0.113** | **-0.059** | **-0.121** | **-0.102** |

^*^ The p-value was less than 0.05.

WT=wall thickness; %WA=wall area percentage; ADMIRE=advanced modeled iterative reconstruction. B1 is the proximal section of the left lower lobe bronchus. B2-B7 are the proximal measurable section of each successive segment downward bronchi of the posterobasal segment of the left lower lobe.

**Appendix Table 2.** The mean and standard deviation of signal-to-noise ratio (SNR) and noise in four kV settings

|  | **ADMIRE**  **level** | **dose** | **Mean±SD** | | | | |
| --- | --- | --- | --- | --- | --- | --- | --- |
|  |  |  | **Standard**  **dose** | **70kV** | **80kV** | **90kV** | **Sn100kV** |
| **SNR** | **ADMIRE 1** | **1/4 dose** | **121.13±16.26** | **74.32±7.79** | **71.33±11.44** | **78.03±10.11** | **76.27±22.22** |
|  |  | **1/10 dose** |  | **43.78±6.73** | **45.31±4.07** | **46.22±14.65** | **46.91±6.23** |
|  |  | **1/20 dose** |  | **39.79±8.84** | **38.07±7.28** | **—** | **33.71±4.43** |
|  | **ADMIRE 3** | **1/4 dose** | **167.57±32.98** | **100.34±9.10** | **99.28±18.21** | **109.02±17.96** | **109.54±32.19** |
|  |  | **1/10 dose** |  | **58.78±11.65** | **60.80±7.94** | **65.11±25.89** | **63.65±8.09** |
|  |  | **1/20 dose** |  | **55.51±16.23** | **50.70±12.84** | **—** | **44.68±5.36** |
|  | **ADMIRE 5** | **1/4 dose** | **242.02±60.76** | **138.91±5.39** | **145.71±29.91** | **162.57±37.37** | **168.11±49.67** |
|  |  | **1/10 dose** |  | **85.60±17.66** | **89.48±15.95** | **96.79±43.42** | **92.99±10.22** |
|  |  | **1/20 dose** |  | **84.49±28.62** | **72.85±23.79** | **—** | **65.39±10.35** |
| **NOISE** | **ADMIRE 1** | **1/4 dose** | **8.24±1.09** | **13.26±1.27** | **14.12±2.62** | **12.80±1.90** | **13.60±3.21** |
|  |  | **1/10 dose** |  | **22.72±2.97** | **21.74±2.05** | **22.74±5.92** | **21.21±2.72** |
|  |  | **1/20dose** |  | **25.41±4.38** | **26.58±5.09** | **—** | **29.54±3.94** |
|  | **ADMIRE 3** | **1/4 dose** | **6.05±1.17** | **9.80±0.81** | **10.23±2.28** | **9.27±1.85** | **9.49±2.31** |
|  |  | **1/10 dose** |  | **17.10±2.88** | **16.34±2.32** | **16.68±5.12** | **15.61±1.92** |
|  |  | **1/20 dose** |  | **18.56±3.92** | **20.38±5.08** | **—** | **22.28±3.00** |
|  | **ADMIRE 5** | **1/4 dose** | **4.28±1.13** | **7.04±0.27** | **7.01±1.64** | **6.38±1.83** | **6.22±1.66** |
|  |  | **1/10 dose** |  | **11.80±2.29** | **11.28±2.35** | **11.56±4.05** | **10.65±1.17** |
|  |  | **1/20 dose** |  | **12.37±2.95** | **14.72±4.95** | **—** | **15.36±2.73** |

SD=standard deviation; ADMIRE= advanced modeled iterative reconstruction; SNR=signal-to-noise ratio

**Appendix Table 3.** Pairwise comparisons of signal-to-noise ratio (SNR) and noise in four kV settings.

| **ADMIRE**  **level** | | **dose** | **p-value** | | | | | |
| --- | --- | --- | --- | --- | --- | --- | --- | --- |
|  |  |  | **70kV vs 80kV** | **70kV vs 90kV** | **70kV vs Sn100kV** | **80kV vs 90kV** | **80kV vs Sn100kV** | **90kV vs Sn100kV** |
| **SNR** | **ADMIRE**  **1** | **1/4 dose** | **0.748** | **0.691** | **0.834** | **0.474** | **0.596** | **0.850** |
|  |  | **1/10 dose** | **0.825** | **0.724** | **0.651** | **0.895** | **0.817** | **0.921** |
|  |  | **1/20 dose** | **0.792** | **－** | **0.359** | **－** | **0.509** | **－** |
|  | **ADMIRE**  **3** | **1/4 dose** | **0.945** | **0.573** | **0.550** | **0.527** | **0.506** | **0.973** |
|  |  | **1/10 dose** | **0.876** | **0.625** | **0.706** | **0.738** | **0.825** | **0.910** |
|  |  | **1/20 dose** | **0.997** | **－** | **0.770** | **－** | **0.748** | **－** |
|  | **ADMIRE**  **5** | **1/4 dose** | **0.797** | **0.374** | **0.276** | **0.525** | **0.400** | **0.834** |
|  |  | **1/10 dose** | **1.000** | **1.000** | **0.997** | **1.000** | **1.000** | **1.000** |
|  |  | **1/20 dose** | **0.616** | **－** | **0.414** | **－** | **0.748** | **－** |
| **NO**  **I**  **S**  **E** | **ADMIRE**  **1** | **1/4 dose** | **0.538** | **0.746** | **0.803** | **0.351** | **0.713** | **0.567** |
|  |  | **1/10 dose** | **0.653** | **0.990** | **0.488** | **0.644** | **0.805** | **0.480** |
|  |  | **1/20 dose** | **0.644** | **－** | **0.116** | **－** | **0.251** | **－** |
|  | **ADMIRE**  **3** | **1/4 dose** | **0.704** | **0.644** | **0.787** | **0.403** | **0.517** | **0.848** |
|  |  | **1/10 dose** | **0.693** | **0.828** | **0.441** | **0.859** | **0.704** | **0.655** |
|  |  | **1/20 dose** | **0.433** | **－** | **0.121** | **－** | **0.417** | **－** |
|  | **ADMIRE**  **5** | **1/4 dose** | **0.972** | **0.472** | **0.371** | **0.494** | **0.390** | **0.857** |
|  |  | **1/10 dose** | **1.000** | **1.000** | **0.998** | **1.000** | **1.000** | **1.000** |
|  |  | **1/20 dose** | **0.269** | **－** | **0.164** | **－** | **0.758** | **－** |

s

ADMIRE= advanced modeled iterative reconstruction; SNR=signal-to-noise ratio

**Appendix Table 4.** Multiple comparisons among the three advanced modeled iterative reconstruction (ADMIRE) levels of signal-to-noise ratio (SNR) and noise.

|  |  | **Multiple comparisons** | | |
| --- | --- | --- | --- | --- |
|  |  | **ADMIRE 1 vs 3** | **ADMIRE 3 vs 5** | **ADMIRE 1 vs 5** |
| **SNR** | **p** | **0.170** | **0.023** | **0.001** |
|  | **95%CI** | **-44.30, 3.63** | **-70.55, 0.46** | **-88.72, -22.03** |
| **Noise** | **p** | **0.014** | **0.029** | **0.000** |
|  | **95%CI** | **1.13, 9.41** | **0.52, 8.81** | **5.80, 14.08** |

CI=confidence interval; ADMIRE= advanced modeled iterative reconstruction; SNR=signal-to-noise ratio
